# Geochronology and geochemistry data for the Elbrus, Tyrnyauz, and Chegem magmatic centers, Greater Caucasus, Russia

**DOI:** 10.1016/j.dib.2021.106896

**Published:** 2021-02-20

**Authors:** I.N. Bindeman, J.-F. Wotzlaw, R.A. Stern, M. Chiaradia, M. Guillong, D.P. Colón

**Affiliations:** a Earth Sciences, University of Oregon, Eugene, OR, USA; b Fersman Mineralogical Museum, Moscow, Russia; c Institute of Geochemistry and Petrology, ETH Zurich, Switzerland; d Canadian Center for Microanalysis, University of Alberta, Canada; e Section des Sciences de la Terre et de L'Environnement, University of Geneva, Switzerland

**Keywords:** Caucasus, Eldjurta granite, Zayukovo, Tyrnyauz, Elbrus, Zircon, Oxygen isotopes, Hydrogen isotopes

## Abstract

The dataset presented here is associated with the article “Young Silicic Magmatism of the Greater Caucasus, Russia with implication for its delamination origin based on zircon petrochronology and thermomechanical modeling” [1]. We present detailed sample descriptions and source locations for the rocks from the Chegem, Tyrnyauz, and Elbrus volcanic center localities presented in that study. The dataset presents extensive isotope and trace element geochemistry of zircon crystals from these rocks, major phenocrysts, and whole rock O and H isotopic and elemental compositions. Zircon ages, trace element compositions, and Hf and O isotopic compositions were obtained by both laser ablation ICP-MS and secondary ionization mass spectrometry in situ techniques and chemical abrasion isotope dilution-thermal ionization mass spectrometry techniques. We also present whole-rock major element compositions obtained by X-ray fluorescence and trace element compositions obtained by solution inductively-coupled plasma mass spectrometry. We also report δ^18^O analyses of phenocrysts and groundmass in samples, δ^18^O-δ^13^C analyses of limestones and limestone xenoliths in the Chegem ignimbrite, and coupled δ^18^O-δD-Δ^17^O analyses of glass and groundmass of rock samples from the Chegem ignimbrites that show abundant evidence of post-emplacement interaction with meteoric waters. To supplement the associated study [1], this article also includes field photographs, cathodoluminescence images of zircons, plots of trace element compositions in zircon, plots of stable isotopic variations in Chegem ignimbrites vs. stratigraphy, and selected trace elemental whole-rock diagrams.

## Specifications Table

**Table:** 

Subject	Earth surface processes Economic geology Geochemistry and petrology
Specific subject area	Magmatism of collision zones, zircon geochronology, oxygen and hydrogen isotope geochemistry, Hf isotopes
Type of data	Tables and plots Field photographs
How data were acquired	Microscope, Laser fluorination-gas source mass spectrometry (MAT253, University of Oregon), TCEA, Gasbench (O-H-C isotopes, U of Oregon), XRF+ ICP-MS (Pomona College, Irkutsk Institute of Geochemistry), SIMS (SHRIMP-RG for U-Pb in zircons, Stanford University), Thermo Triton Plus mass spectrometer (U-Pb in zircons ETH Zürich), Thermo Element XR sector field ICP-MS, (trace elements in zircons, ETH Zürich), photon laser ablation system coupled to a Thermo Neptune Plus MC-ICP-MS (Hf isotpes in zircons, University of Geneva)
Data format	Raw Analyzed Filtered
Parameters for data collection	Analytical conditions varied depending on the method used and are described individually
Description of data collection	Sample coordinates and description, XRF and ICP-MS analyses of rocks, LA-ICPMS analyses of zircons for U-Pb age, SHRIMP analyses of zircons for U-Pb age, CA_IDTIMS analyses of zircons for U-Pb age, LAICPMS analyses of zircons for Hf isotopes, SIMS analyses of zircons for O isotopes, laser fluorination analyses of phenocrysts and groundmass samples for O isotopes, TCEA analyses of glass and groundmass samples for H isotopes, laser fluorination analyses of samples for triple O isotopes, Gasbench analyses of limestone, skarn and carbonaceous silicates for O and C isotopes.
Data source location	Institution: University of Oregon, Earth Sciences, Eugene, OR, USA Latitude and longitude (GPS coordinates) for collected samples: Latitude:42°30′ to 43°30′ Longitude: 43°16′ to 43°64′ Secondary data: C. Gazis study of Chegem ignimbrite [2], [3], [4]. O isotopes of groundmass samples used in conjunction with our analyses; we reanalysed some samples used in [3].
Data accessibility	Data is hosted with this article as supplementary
Related research article	I.N. Bindeman, D.P. Colón, J.-F. Wotzlaw, R. Stern, M. Chiaradia, M. Guillong, Young Silicic Magmatism of the Greater Caucasus, Russia with implication for its delamination origin based on zircon petrochronology and thermomechanical modeling, Journal of Volcanology and Geothermal Research, https://doi.org/10.1016/j.jvolgeores.2021.107173

## Value of the Data

• This is a large new chemical and isotopic analytical dataset for young silicic rocks of the Greater Caucasus.
• This includes a large and targeted zircon U-Pb, and O and Hf isotopic zircon dataset for Chegem, Elbrus and Tyrnyauz.
• This study adds large new H and O isotopes and water concentrations for ignimbrites of Chegem and C isotopes in carbonate xenoliths, documenting secondary alteration and post-emplacement interaction of tuff with xenoliths.

## 1 Data Description

The data presented here include whole-rock chemical analyses of 40 samples of young volcanic rocks from the Greater Caucasus for major and trace elements (Supplementary Table 4), oxygen isotopic composition of rocks and minerals (Supplementary Table 3), hydrogen and carbon isotopic composition of rocks and glasses (Supplementary Table 3), zircon geochronology from 9 samples including CA-IDTIMS for 3 samples, SHRIMP for 2 samples, and LA-ICP_MS for 6 samples (Supplementary Tables 2 and 6). It also presents LA-ICPMS analyses of trace element variations in dated zircons, as well as in situ Hf and O isotopes in these zircons (Supplementary Tables 2 and 3). We also present field observations in the form of measured sections and field photographs presented here and in the associated research article.

Supplementary Table 5 provides a list of samples that were used in this study and lists the methods that were used to study them. It also provides GPS coordinates and field (outcrop) description of the place of their collection.

Supplementary Table 6 provides results of CA_ID-TIMS investigation (precise U-Pb dating) of a subset of three zircon populations from the Chegem Caldera, top and the bottom ignimbrites and a granite porphyry. It also provides in situ analyses of core and rims for U-Pb age and trace elements as obtained by LA-ICP-MS for the same crystals, later dated for CA-IDTIMS.

Supplementary Table 7 provide results of oxygen isotope investigation of samples by laser fluorination targeting phenocrysts, groundmass and whole-rock samples. Two most δ^18^O-depleted samples of hydrothermally-altered groundmass were additionally studied for triple oxygen isotopes to investigate effects of water-rock interaction and derive −13‰ value for altering meteoric water (Fig. A3 in ref. [1]). It also reports results of D/H + H_2_O investigation of groundmass, vitropyric glass and whole rocks by TCEA to investigate effects of secondary hydration by meteoric waters that are plotted in Fig. A2 of ref. [1]. It additionally reports results of C and O isotopes in carbonate bearing samples of limestone xenoliths by Gasbench to investigate effects of decarbonation and results are plotted in Fig. A1 if ref. [1]. All analyses were performed at the University of Oregon Stable Isotope Lab.

Supplementary Table 1 provides results of LA-ICP-MS dating of zircons and measurements of trace element concentrations in the same spot for all dated sample of this work, including ignimbrites and lavas from Elbrus, Chegem, Zayukovo, and Tyrnyauz. It also lists Ti concentrations and Ti-in-zircon temperatures, U and Th concentrations, as well as Eu anomalies that are plotted in Figs. 2 and 3.

**Fig. 1:**
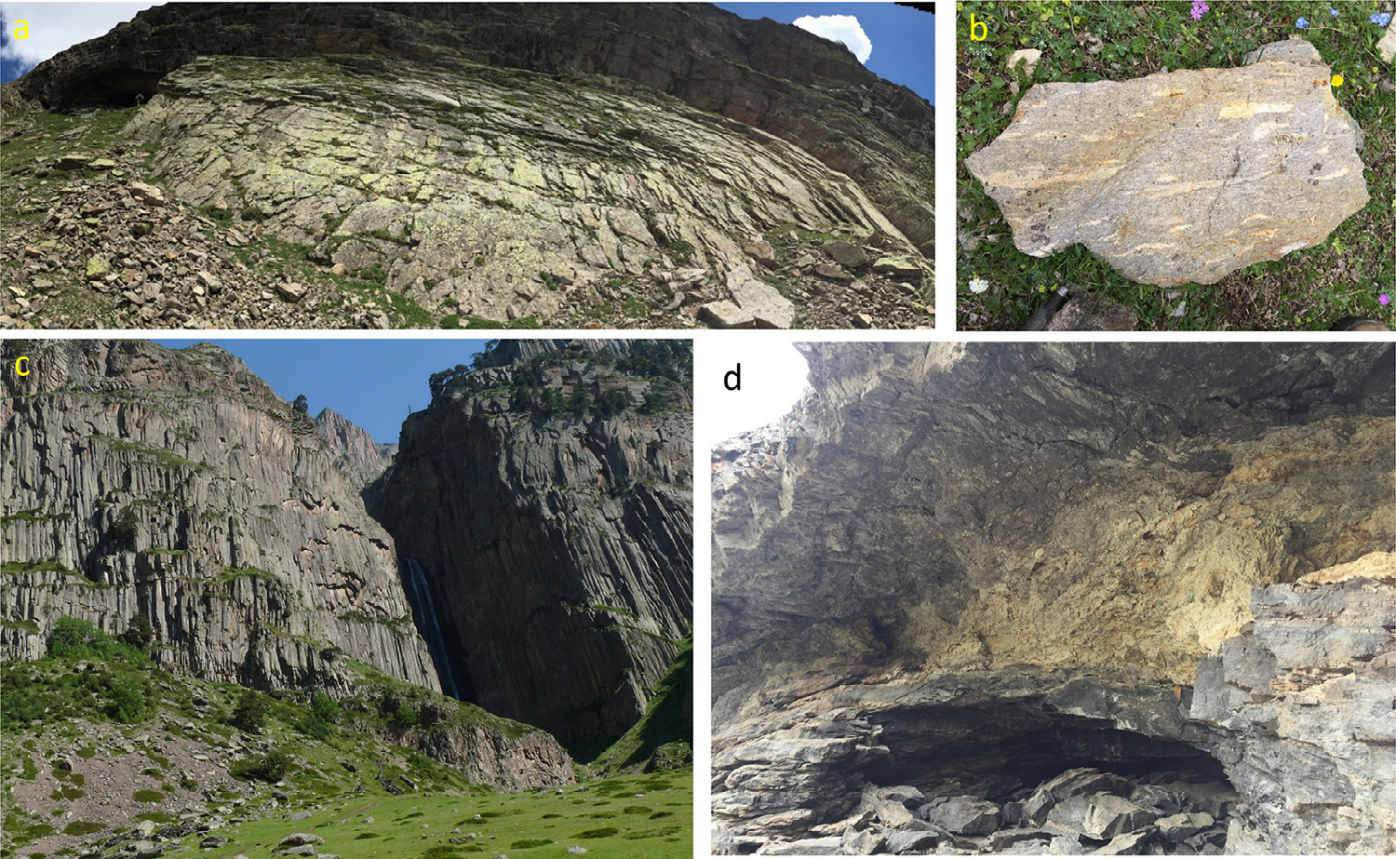
Field relations in the middle-upper portion of intracaldera Chegem Ignimbrite. Dome-like structure in the western part (a), welded ignimbrite with fiamme (b), Columnar joints in the southern part c), and arch-like bubble expansion with alternating perlite-carbonate-rich core, western part.

**Fig. 2:**
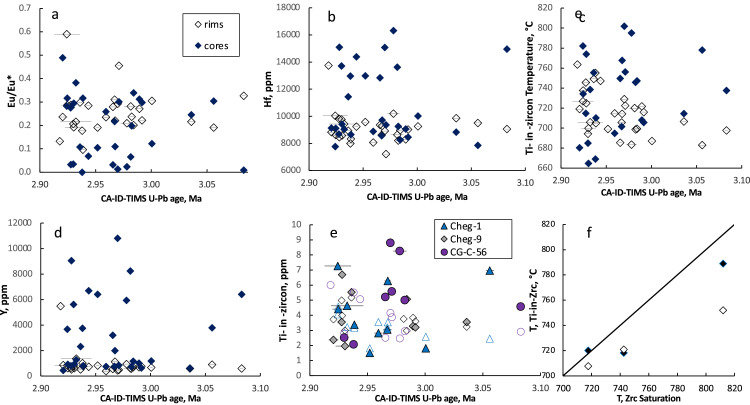
Evolution of trace element concentrations and ratios from cores to rims of zircons (measured by LA-ICP-MS) vs. ID-TIMS ages **(a-e)** and zircon thermometry **(c,f)**. Age uncertainty is within symbols unless shown. Notice stronger heterogeneity of cores vs. rims but an overall lack of trends. Ti-in-zircon temperatures are lower than zircon saturation temperatures **(f)** and rims show an overall increase in temperature with youth explained by their growth during heating upon reservoir assembly. Data is presented in the Supplementary Table 7 of this paper and further trace elemental plots in zircons are presented in Fig. 9 of the associated research paper.

**Fig. 3:**
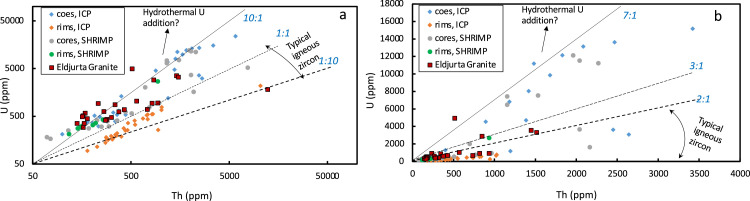
U and Th in zircon from Chegem and Eldjurta samples measured by LA-ICP-MS and SHRIMP, showing high-U variable U/Th ratios. Panel a) has log Y axis, while panel b) has a linear scale. Data is presented in the Supplementary Tables 4, 6, and 7 of this paper and further trace elemental plots in zircons are presented in Fig. 9 of the associated research paper [1].

**Fig. 4:**
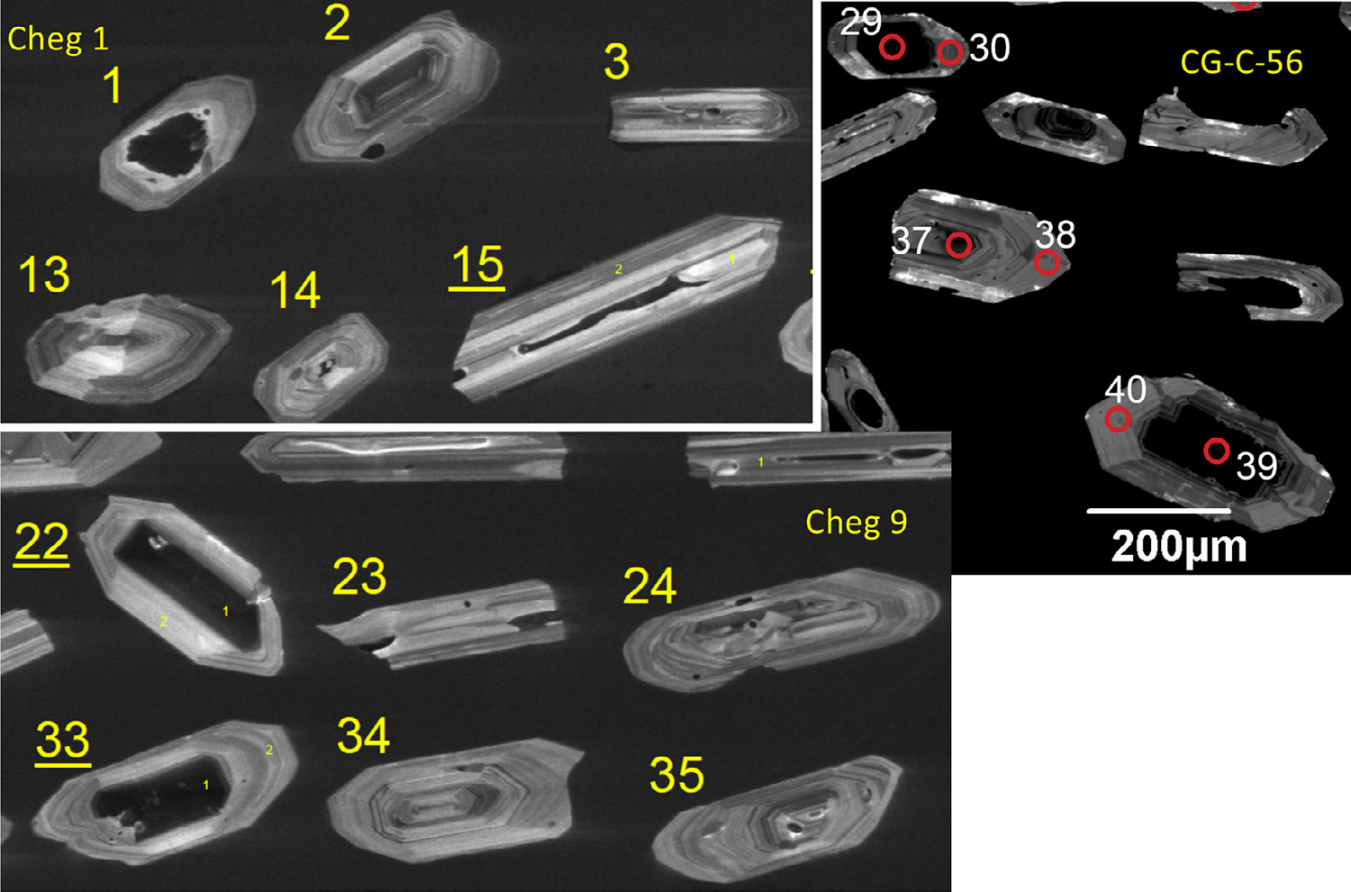
Cathodoluminescence images of selected zircons from sample Cheg-1 (bottom ignimbrite), Cheg-9 (top ignimbrite), and CG-C-56 (granite porphyry) indicating predominance of gray oscillatory rims, and frequent very dark, U-rich cores. These zircon types are present across the entire 3.05–2.9 Ma crystallization history of zircon in Chegem magmas, as well as in other studied Greater Caucasus ignimbrites. Circles in the image of CG-C-56 zircons indicate locations of SIMS analyses (see Supplementary Table 2).

**Fig. 5:**
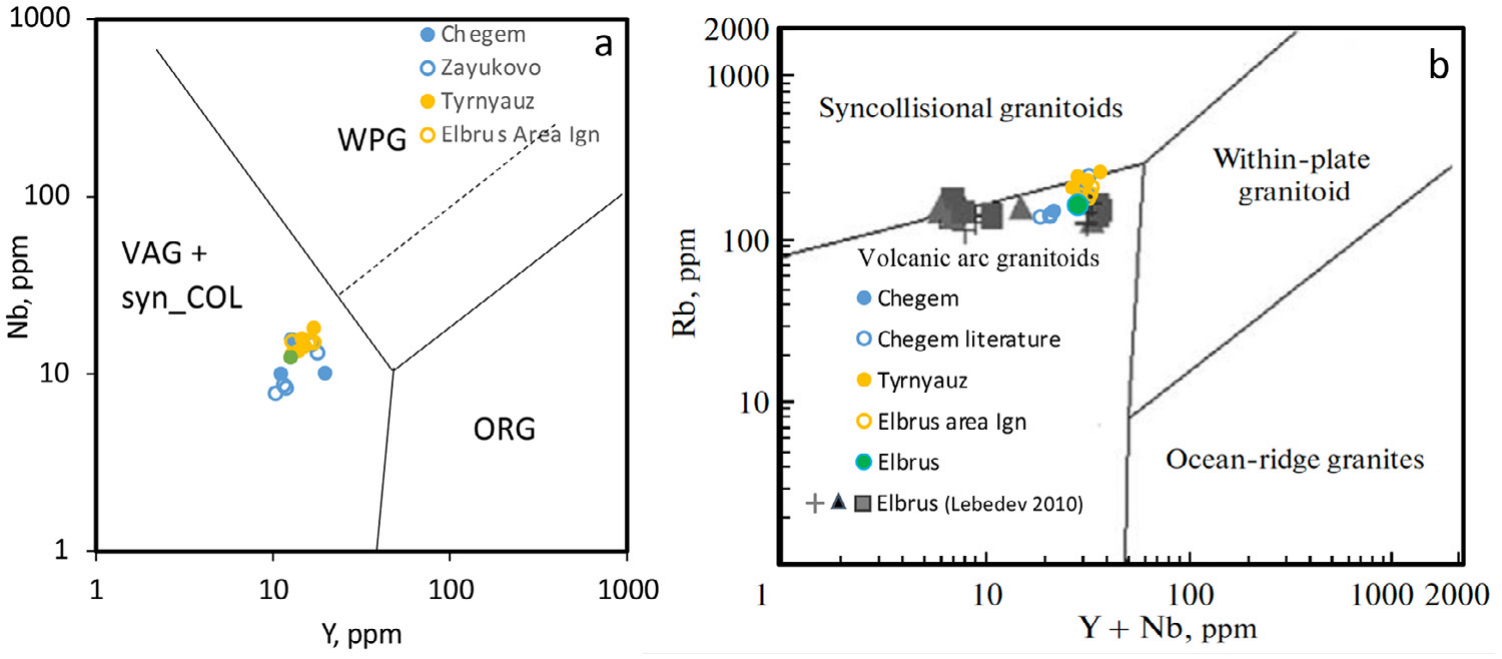
Whole rock compositions of Caucasus magmas plotted on the discrimination diagrams of [17]. Extra data for Elbrus plotted in (b) is sourced from reference [18]. Supplementary Table 4.

**Fig. 6:**
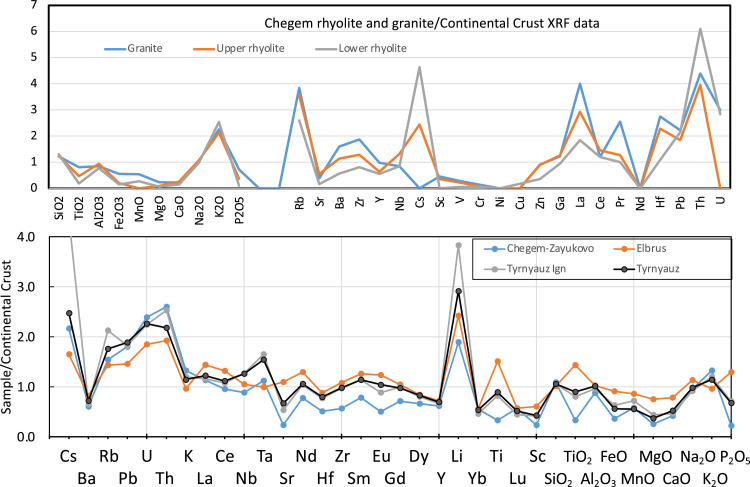
Spidergrams for Chegem (top) and other Caucasus magmas (bottom) normalized to the average composition of the continental crust [19]. Data is from Supplementary Table 4.

Supplementary Table 2 provides results of SHRIMP U-Pb dating, trace elements, and same spot co-registered oxygen SIMS isotope values, and epsilon Hf isotope analyses (by LAICPMS) made in three mounts of zircons extracted from nine samples of Elbrus, Chegem, Zayukovo, and Tyrnyauz areas. Examples of zircons that were analyzed are given in Fig. 4. Data from these Table are plotted in Figs. 4–9 of the associated research paper [1].

Supplementary Table 3 provide raw and reduced data for LAICPMS analyses of Hf isotopes of zircons, also listed in the supplementary Table 2, and studied for U-Pb age using LA-ICP-MS (Supplementary Table 7) performed at the University of Geneva. Data for concurrently run standards is below.

Supplementary Table 4 presents XRF and ICPMS analyses of major and trace elements of studied samples of ignimbrites and lavas, used for dating and other types of analyses, color-coded by expected source/region of origin as is determined by U-Pb dating and O-Hf isotopic fingerprinting (see associated research paper [1] for discussion. Elbrus-area ignimbrites, for example, are color-coded to originate from the Tyrnyauz area. Data is plotted on discrimination diagrams (Fig. 5) and spidergrams (Fig. 6).

## 2 Experimental Design, Materials and Methods

### 2.1 Fieldwork and sampling

We sampled the most prominent ignimbrites on the northern slopes of modern Elbrus edifice, at Tuzluk Mt. (sample ELB-1), and the Lower (ELB-13, −12) and Upper (ELB-9, −11) Stone Mushroom ignimbrites near the upper Birdjalisu river [1; Fig. 2 within, Supplementary Table 5]. Also at Elbrus, we sampled an additional 10 lavas of varying composition (other samples in ELB 2–19). In Tyrnyauz, we sampled both the Eldjurta granite (ELB-20) and a rhyolite (ELB-21) cross-cutting it. At Chegem, we sampled transects of intracaldera tuff (samples CHEG-1 and CHEG-9 targeted for zircon), the intracaldera porphyritic intrusion (CG-C-56), and a sample of the outlying Zayukovo Tuff (ELB-23). Our study also utilized samples from the study of [2], stored at the Caltech sample repository, for our whole-rock oxygen, hydrogen, and carbon isotope analyses (below). See [1] for further details of our sampling strategy. Figs. 1 and 3 in the associated research paper present observations.

### 2.2 Whole rock chemical composition

Whole rock major and trace element analyses were performed at the Irkutsk Institute of Geochemistry by XRF and solution ICP-MS (A.B. Perepelov, lab director), and at Pomona College (J.S. Lackey, lab director). XRF methods included fusion of ∼1–5 g of sample with Li-tetraborate fuse. USGS rock standards were used for cross checking the data. Supplementary Table 4 presents these data.

### 2.3 Stable isotopes in major phenocrysts and groundmass/glasses

Oxygen isotope measurements on phenocrysts and groundmass were performed by laser fluorination and gas-source mass spectrometry at the University of Oregon, following the methods [5]. For laser fluorination of phenocrysts, we use a regular set up while for groundmass or glass we used an airlock sample chamber and samples were introduced one by one, prior to fluorination. We used BrF_5_ as a reagent and boiling Hg diffusion pump to get rid of excess of F_2_ gas, then converted purified O_2_ into CO_2_ and run it in a dual inlet mode on MAT253 mass spectrometer, integrated with the vacuum line. Samples yields were measured in a calibrated volume using a Baratron gage. UWG2 garnet (δ^18^O=5.80‰, [20]) and UOG garnet (δ^18^O=6.52‰ [5], calibrated relative to UWG2) were used to calibrate the data on VSMOW scale. Errors of individual analyses is better than ±0.1‰ for regular set up and better than ±0.1–0.15‰ for an airlock. For triple oxygen isotope analyses of two groundmass samples and the SCO standard we used an airlock sample chamber, and generated O_2_ gas was put through the 8 ft long gas chromatographic column for its purification from NFx compounds. Then it was run as O_2_ gas 5 timesx8 cycles in a dual inlet mode, against a well calibrated oxygen gas standard. In the same lab, we also made additional analyses of C and O isotopes in carbonates using a Thermo Fisher Gasbench system, using acidification of samples with orthophosphoric acid and He flush prior to continuous flow analysis of generated CO_2_ gas on MAT253. We used TCEA (high-temperature-conversion, elemental analyzer) coupled with MAT253 isotope ratio mass spectrometer for H isotopes and total H_2_O simultaneously from a multitude of groundmass and glass samples for δ_D_ (see [6]) in a continuous flow mode. Mica standards USGS57 (biotite) and USGS58 (muscovite) are used for calibration and are analyzed 3–5 times throughout each analytical session, commonly also with an internal laboratory biotite standard, BUD. All isotope compositions are expressed the attached tables in delta notation relative to Vienna Standard Mean Ocean Water (VSMOW for O and H isotopes), and PDB for C isotopes. See [7] for more details about the thermal decomposition and pyrolysis method. Supplementary Table 7 presents these data.

### 2.4 Zircon geochemistry and U-Pb dating

Zircon grains were imaged via cathodoluminescence and scanning electron microscope (Fig. 4) prior to isotopic and chemical analysis. Details of analytical methods are provided in [1]. Three epoxy mounts were prepared. Mount #1, which had zircons from the Chegem center, was analyzed for δ^18^O via the Cameca 1280 ion microprobe (SIMS) instrument at the Canadian centre for Isotopic Microanalysis at the University of Alberta (Richard Stern, analyst, Supplementary Table 2). This mount was then repolished and reanalyzed at the same spots using the SHRIMP_RG instrument at the Stanford/USGS facility (Figs. 5 and 6).

Mount #2 contained more zircons from the same Chegem samples, first annealed at 900 °C for 48 h prior to mountain and polishing. Zircon were measured for trace elements and U/Pb ages at their cores and rims using the ASI Resonetics 155 laser ablation system coupled to a Perkin Elmer NexION 2000 quadrupole ICP-MS at ETH Zürich. A subset of the grains from Mount #2 that had overlapping core and rim ages were plucked from the mount and chemically abraded [8] for 13 h at 180 °C. Zircons were then spiked with Earthtime ^202^Pb-^205^Pb-^233^U-^235^U tracer solution [9] and processed for ID-TIMS analyses using the protocols described in [10], (Supplementary Table 6).

Mount #3 was made with other Caucasus zircons from the Elbrus, Tyrnyauz, and Zayukovo localities [1]. U/Pb ages and trace element compositions were obtained by sector field LA-ICP-MS at ETH Zürich (Supplementary Table 1). Trace element analyses of zircons at adjacent spots was performed employing an ASI Resonetics 155 laser ablation system coupled to a Thermo Element XR sector field ICP-MS at ETH Zürich. Primary reference materials were the same as for the Q-ICP-MS analyses described above. The majority of the ICP-MS dated grains, including Elbrus and Tyrnyauz samples, were also analyzed for Hf isotopes employing a Photon laser ablation system coupled to a Thermo Neptune Plus MC-ICP-MS at the University of Geneva (Supplementary Table 3). Reproducibility of standard zircons over the course of the analyses was precise at ±0.75 epsilon units based on standard reproducibility. Data were reduced off-line using an excel spreadsheet and consisted of blank subtractions, removing the isobaric interference of ^176^Lu and ^176^Yb on mass 176 [11] and correcting the resulting ^176^Hf/^177^Hf ratio for mass bias using an exponential law [12]. βHf and βYb mass bias coefficients were calculated from the measured ^179^Hf/^177^Hf and ^173^Yb/^171^Yb with the reference values of [13] (^179^Hf/^177^Hf=0.7325) and [14] (^173^Yb/^171^Yb=1.1234) respectively. Isobaric interferences of ^176^Yb and ^176^Lu with ^176^Hf were corrected using ^176^Yb/^173^Yb=0.786954 and ^176^Lu/^175^Lu=0.02645 respectively [14]). Time resolved ^176^Hf/^177^Hf plots were used to detect any heterogeneities like inclusions or different domains (e.g., inherited core) with different ^176^Hf/^177^Hf ratios. Only non-perturbed spectra were retained. Initial ^176^Hf/^177^Hf ratios and initial εHf were calculated using the ^206^Pb/^238^U date of the respective crystal, the CHUR parameters of [15] (^176^Hf/^177^Hf = 0.282785 and ^176^Lu/^177^Hf= 0.0336) and λ^176^Lu=1.87 × 10–11 year-1 [16]. Supplementary Tables 1–3 and 6 present these data.

## CRediT Author Statement

**Ilya Bindeman:** Fieldwork, O-C-H isotope analyses, zircon extraction, paper writing and editing; **Dylan Colón:** Writing and editing, interpretation and modeling in [1]; **Jörn-Frederik Wotzlaw:** Zircon ID-TIMS and LA-ICP-MS analysis, data plotting and paper editing; **Richard Stern:** SIMS analysis of zircons for O isotopes; **Massimo Chiaradia:** Hf isotope analysis of zircons by LA-ICP-MS; **Marcel Guillong:** LA-ICP-MS analys s of zircons.

## Declaration of Competing Interest

Authors have no competing interests.

## Appendix Supplementary materials

Image, application 1

Image, application 2

## Footnotes

Supplementary material associated with this article can be found in the online version at doi:10.1016/j.dib.2021.106896.
